# Salvage transmural lithotripsy via endoscopic ultrasound-guided hepaticogastrostomy for basket impaction in Roux-en-Y anatomy

**DOI:** 10.1055/a-2743-2385

**Published:** 2025-11-28

**Authors:** Saburo Matsubara, Tomohiro Arai, Risa Sunada, Keito Nakagawa, Noriko Murakami, Morito Ikeda, Naosuke Kuraoka

**Affiliations:** 1 Department of Gastroenterology and Hepatology, Saitama Medical Center, Saitama Medical University, Kawagoe, Japan

A 70-year-old man with previous distal gastrectomy and Roux-en-Y reconstruction underwent balloon-assisted endoscope-guided endoscopic retrograde cholangiopancreatography (BE-ERCP) for choledocholithiasis ( Fig. 1 **a** ). Following papillary large balloon dilation, stone extraction was attempted using a wire-guided retrieval basket (RASEN2, Kaneka Medix, Osaka, Japan), but basket impaction occurred ( Fig. 1 **b** ). The basket catheter was cut proximal to the handle, and the outer sheath was removed through the working channel. The endoscope (EI-580BT; Fujifilm, Tokyo, Japan) was withdrawn while maintaining the over tube, remnant basket wire, and guidewire in position. After insertion of a SpyScope DS II (Boston Scientific, Marlborough, Massachusetts, USA) over the guidewire through the over tube ( Fig. 1 **c** ), electrohydraulic lithotripsy (EHL) was attempted. However, the SpyScope inadvertently slipped from the bile duct due to complex anatomy. A 5-Fr nasobiliary tube was subsequently placed over the guidewire. Given these technical challenges, endoscopic ultrasound-guided hepaticogastrostomy was performed at segment B3, with bile duct distension achieved by saline injection through the nasobiliary tube. After deployment of a 10-mm × 6-cm fully covered self-expandable metal stent (FCSEMS, HILZO stent; BCM, Gyeonggi-do, Korea; Fig. 2 **a** ), the stent was dilated with an 8-mm balloon catheter, allowing successful SpyScope insertion. Under direct visualization, EHL fragmented the stones within the impacted basket ( Fig. 2 **b, c** ), and the basket catheter was subsequently retrieved successfully ( Video 1 ). The patient developed mild pancreatitis and peritonitis, which resolved with conservative management.

**Fig. 1:**
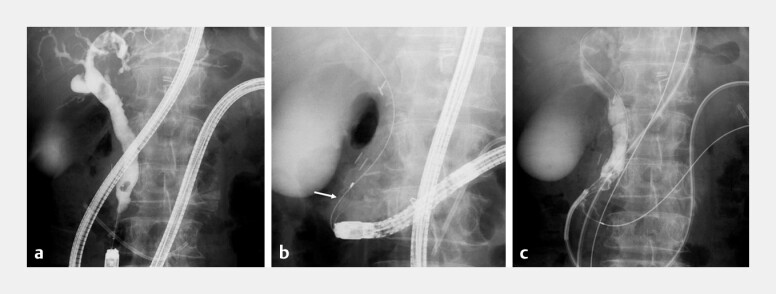
Basket impaction during balloon-assisted endoscopy-guided endoscopic retrograde cholangiopancreatography for common bile duct stones in Roux-en-Y anatomy. **a** A cholangiogram revealed common bile duct stones. **b** Basket impaction occurred with a wire-guided basket catheter (arrow). **c** A SpyScope DS II (Boston Scientific, Marlborough, Massachusetts, USA) was inserted over the guidewire through the over tube.

**Fig. 2:**
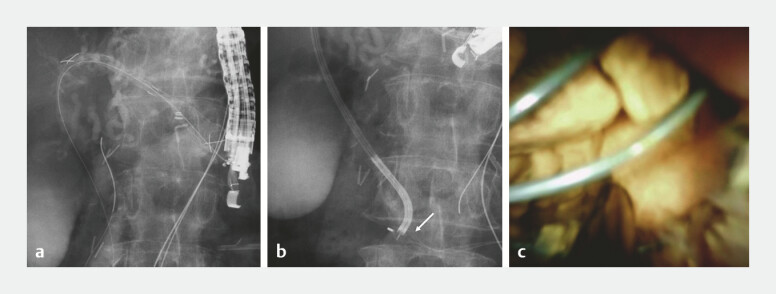
Salvage transmural lithotripsy via endoscopic ultrasound-guided hepaticogastrostomy. **a** A 10-mm × 6-cm fully covered self-expandable metal stent was placed at B3. **b** Electrohydraulic lithotripsy (EHL) of bile duct stones within the impacted basket (arrow) using the SpyScope through the metal stent. **c** Direct visualization of EHL under SpyScope guidance.

Salvage transmural lithotripsy via endoscopic ultrasound-guided hepaticogastrostomy for basket impaction in Roux-en-Y anatomy.Video 1

Basket impaction represents a serious complication during ERCP for choledocholithiasis, with an incidence of approximately 0.3% 1 . While cholangioscopy-guided EHL and laser lithotripsy are established salvage techniques 2 3 , their application in BE-ERCP for patients with Roux-en-Y anatomy remains technically challenging due to limited scope maneuverability and working channel constraints. EUS-guided transmural lithotripsy through an urgently created hepaticogastrostomy with a FCSEMS offers a viable salvage approach for such complex cases 4 .

Endoscopy_UCTN_Code_CPL_1AK_2AC

## Notes

Endoscopy E-Videos https://eref.thieme.de/e-videos
*E-Videos* is an open access online section of the journal *Endoscopy* , reporting on interesting cases and new techniques in gastroenterological endoscopy. All papers include a high-quality video and are published with a Creative Commons CC-BY license. Endoscopy E-Videos qualify for HINARI discounts and waivers and eligibility is automatically checked during the submission process. We grant 100% waivers to articles whose corresponding authors are based in Group A countries and 50% waivers to those who are based in Group B countries as classified by Research4Life (see: https://www.research4life.org/access/eligibility/ ). This section has its own submission website at https://mc.manuscriptcentral.com/e-videos .
